# HER2 status in recurrent/metastatic androgen receptor overexpressing salivary gland carcinoma patients

**DOI:** 10.3389/fonc.2022.1096068

**Published:** 2023-01-17

**Authors:** Stefano Cavalieri, Imperia Nuzzolese, Arianna Ottini, Cristiana Bergamini, Carlo Resteghini, Elena Colombo, Salvatore Alfieri, Pasquale Quattrone, Giuseppina Calareso, Nicola Alessandro Iacovelli, Marzia Franceschini, Lisa Licitra

**Affiliations:** ^1^ Head and Neck Medical Oncology Department, Fondazione IRCCS Istituto Nazionale dei Tumori, Milan, Italy; ^2^ Department of Oncology and Hemato-Oncology, University of Milan, Milan, Italy; ^3^ Pathology Department, Fondazione IRCCS Istituto Nazionale dei Tumori, Milan, Italy; ^4^ Radiology Department, Fondazione IRCCS Istituto Nazionale dei Tumori, Milan, Italy; ^5^ Radiotherapy Department, Fondazione IRCCS Istituto Nazionale dei Tumori, Milan, Italy

**Keywords:** HER2, androgen receptor, SDC, salivary duct carcinoma, SGC, salivary gland carcinoma, brain metastasis

## Abstract

**Background:**

Overexpression of human epidermal growth factor receptor type 2 (HER2) occurs in almost 25-30% of androgen receptor (AR)-positive salivary gland carcinomas (SGCs), notably salivary duct carcinoma (SDC) and adenocarcinoma not otherwise specified (NOS). In the last years, several studies have reported the clinical benefit of HER2 directed therapies in this setting. This work aims at describing the natural history of AR-positive recurrent/metastatic (R/M) SGC patients, based on HER2 amplification status.

**Methods:**

Consecutive R/M AR-positive SGC patients accessing our Institution from 2010 to 2021 were analyzed. Descriptive statistics and survival analyses were performed to present the clinical characteristics of the selected patients and the outcomes, based on HER2 status. A specific focus was dedicated to patients developing metastases to the central nervous system (CNS).

**Results:**

Seventy-four R/M AR-positive SGC patients (72 men) were analyzed. Median follow-up was 36.18 months (95% CI 30.19-42.66). HER2 status was available in 62 cases (84%) and in 42% the protein was overexpressed (HER2+). Compared with patients with HER2- SGCs, in patients with HER2+ disease, HR for disease recurrence was 2.97 (95% CI 1.44-6.1, p=0.003), and HR for death from R/M disease was 3.22 (95% CI 1.39-7.49, p=0.007). Moreover, the HER2+ group showed a non-significant trend towards a higher prevalence of CNS metastases (40% vs. 24%, p=0.263). Patients developing CNS metastases had shorter survival than those who did not; at bivariate analysis (covariates: CNS disease and HER2 status), HER2 status demonstrated its independent prognostic significance.

**Discussion:**

In our patient population, HER2 amplification was a negative prognostic factor, and it was associated with a non-statistically significant higher risk of developing CNS metastasis. Further studies are needed to explore the potential clinical benefit of tackling the two biological pathways (AR and HER2) in patients affected by this rare and aggressive malignancy.

## 1 Introduction

Epithelial malignancies arising from the salivary glands (SGCs, salivary gland carcinomas) are rare neoplasms. More than 20 entities are included in the World Health Organization (WHO) classification (1). Specific pathologic types, notably salivary duct carcinoma (SDC) and adenocarcinoma not otherwise specified (NOS) may overexpress androgen receptors (AR). A fraction (average 25-30% up to approximately 40%, depending on the published case series (2, 3) of AR-positive SGCs are characterized by human epidermal growth factor receptor type 2 (HER2) amplification (HER2-positive) (4). AR overexpression is almost definitional in SDCs, and consistently AR-negative SDCs are very rare and this diagnosis should be regarded with skepticism (4). Given that the vast majority of HER2-positive SGC have a SDC histology, the present study is focused on two cohorts: AR-positive HER2-positive; AR-positive HER2-negative.

Similarly to other cancer types, also in SGC both AR and HER2 may be targeted by hormone therapy (5–7), and anti-HER2 agents as trastuzumab (8, 9), trastuzumab plus pertuzumab (10), ado-trastuzumab emtansine (11, 12), trastuzumab deruxtecan (13).

From a prognostic point of view, despite HER2-overexpressing SDCs are known to have worse outcomes than HER2-negative cases (14, 15), their natural history is still unknown. Moreover, both patients with HER2-positive breast and HER2-positive gastric cancers, showed a higher incidence of distant metastases located in the central nervous system (CNS) (16, 17), but in HER2-positive SGCs we lack an in-depth analysis of this feature.

The description of a case series of AR-positive SGCs with available HER2 status may provide further knowledge on this topic.

## 2 Methods

This was a retrospective observational study aimed at describing the natural history of R/M AR-positive SGC according to HER2 status, with a particular focus on patients with CNS metastases, defined as any distant site at any level of the CNS – including carcinomatous meningitis – deemed unequivocal at clinical and radiological level.

We identified consecutive R/M AR-positive SGC patients accessing our Institution from 2010 to 2021, and we selected cases with availability of HER2 status. HER2 was considered positive when immunohistochemistry (IHC) score was 3+, or 2+ confirmed by an *in situ* hybridization (ISH). Cases with 0, 1+ or 2+ with a negative ISH were considered HER2-negative (3).

For the analysis of the prevalence of CNS metastases, subjects with unavailable HER2 status were included as well, but they were analyzed separately. In all cases, the pathologic diagnosis was reviewed and confirmed by an expert pathologist [PQ] dedicated to the diagnosis of rare head and neck cancers, with more than 20 years of experience in the field.

The following clinical variables were collected: gender, age, histology (SDC, adenocarcinoma NOS) AR status (positive, weak), HER2 status (positive, negative), CNS metastases (present, absent), timing of brain metastases (at primary diagnosis – defined as diagnosed within 3 months from the diagnosis of primary disease – or after therapies), previous treatments (loco-regional therapies for primary disease, treatments for R/M disease), treatments for CNS metastases.

The following time-dependent variables were recorded: disease-free interval (DFI, defined as the interval time between the date of primary tumor diagnosis and the date of R/M disease diagnosis), only for cases without metastatic disease at diagnosis of primary tumor (i.e., for the DFI calculation, subjects with metastatic disease at diagnosis were excluded); CNS-metastasis free survival (CNSmfs), only for cases without CNS metastases at diagnosis of primary tumor; time to first CNS metastasis (TTCNS), only for cases without CNS metastases at diagnosis of primary tumor; overall survival (OS) measured from 3 different timepoints:

OS(a) from primary disease, defined as the interval time between the date of primary tumor diagnosis and the date of death or last follow-up;
OS(b) from R/M disease, defined as the interval time between the date of R/M disease diagnosis and the date of death or last follow-up;
OS(c) from CNS metastases onset, defined as the interval time between the date of CNS metastases diagnosis and the date of death or last follow-up, only for cases with CNS metastases.

Data cut-off date was 31/12/2021. Median follow-up, with the respective 95% confidence interval (CI) was estimated through reverse Kaplan-Meier method, measuring the interval time from the date of R/M diagnosis to the date of death or last follow-up. Hazard ratios (HRs) were estimated with Cox proportional hazard model.

Patients with CNS metastases were classified in four groups according to the disease presentation:

1) upfront CNS disease (interval between primary and CNS disease diagnosis ≤ 3 months),
2) CNS metastases after metastatic disease at diagnosis (DFI ≤ 3 months and interval between metastatic and CNS disease > 3 months),
3) CNS metastases as first disease recurrence after treatment for loco-regional disease (DFI > 3 months and interval between metastatic and CNS disease ≤ 3 months),
4) CNS metastases as subsequent recurrence after palliative treatments for R/M disease diagnosed after treatment failure for loco-regional primary disease (DFI > 3 months and interval between metastatic and CNS disease > 3 months).
Descriptive statistics were provided for the main clinical characteristics of each of these four groups.

Descriptive statistics were performed to present the clinical characteristics of the selected patients. To analyze contingency tables Fisher’s exact or chi-squared tests were used, as appropriate.

Time-dependent variables were estimated with Kaplan-Meier method and compared with log-rank test. Multivariable analyses were performed with Cox proportional hazard methods. Statistical analyses were performed using SAS^®^ OnDemand for Academics. Statistical significance was set at 0.05.

The conduction of this retrospective study was approved by the Institutional Ethical Committee on 22/12/2021 (local study identifier INT 270-21).

## 3 Results

### 3.1 Natural history according to HER2 status

We identified 74 R/M AR-positive SGC patients, with a median follow-up of 36.18 months (95% CI 30.19-42.66). HER2 status was available in 62 cases (84%). The prevalence of HER2-positive disease was 42% (26 patients), and the main clinical characteristics of the study population are reported in **Table 1**.

**Table 1:** Patient characteristics.

	General	HER2-positive	HER2-negative	p value
Number	62	26 (42%)	36 (58%)	-
Sex				
M	60 (97%)	25 (96%)	35 (97%)	1.0*
F	2 (3%)	1 (4%)	1 (3%)	
**Median age**	62 years (range 27-78)	57.5 years (range 27-74)	61.5 years (range 39-78)	0.074**
Histologic type				
SDC	51 (82%)	23 (88%)	28 (78%)	0.332*
Adenoca NOS	11 (18%)	3 (12%)	8 (22%)	
Previous condition				
Yes	12 (19%)	4 (15%)	8 (22%)	
RT-induced	8 (13%)	1 (4%)	7 (19%)	
NPC	4	1	3	
Lymphoma	3	–	3	0.746
Other HNSCC	1	–	1	(yes vs. no)*
Ex pleom. ad.	3 (5%)	2 (8%)	1 (3%)	
Pregnancy	1 (2%)	1 (4%)	–	
No	50 (81%)	22 (85%)	28 (78%)	
Treatments				
LR treatment	19 (31%)	11 (42%)	8 (22%)	0.104
None	5 (8%)	3 (12%)	2 (6%)	(yes vs. no)*
Surgery	21 (34%)	6 (23%)	15 (42%)	
Surgery-RT	17 (27%)	6 (23%)	11 (31%)	0.119 (0 vs. ≥ 1)*
Surgery-CRT	7 (11%)	5 (19%)	2 (6%)	
RM disease	20 (32%)	8 (31%)	12 (33%)	0.032 (ADTvs.
Lines of systemic treatments	30 (48%) 5 (8%)	11 (42%) 2 (8%)	19 (53%) 3 (8%)	never ADT)*0.312 (yes vs. no)*
0	49 (79%)	17 (65%)	32 (89%)	
1	36 (58%)	14 (54%)	22 (61%)	
2-3	7 (11%)	7 (27%)	0	
≥4	3 (5%)	0	3 (8%)	
Treatments ADT CT Anti-HER2 Other targeted therapy Palliative RT	23 (37%)	9 (35%)	14 (39%)	
CNS metastases				
Yes	19 (31%)	10 (38%)	9 (25%)	0.278*
No	43 (69%)	16 (62%)	27 (75%)	
**Median survival**				***
DFI	16.33 months	12.11 months	19.28 months	0.002
CNSmfs TTCNS	(95% CI 11.57-19.47) NR (95% 62.9-NR)	(95% CI 5.92-15.19) NR (95% 19.15-NR)	(95% CI 15.56-23.09) NR (95% 62.9-NR)	0.08 0.083
OS from primary	19.87 months	18.02 months	27.8 months	0.0007
OS from R/M	(95% CI 14.8-29.24)	(95% CI 5.92-24.64)	(95% CI 12.04-62.89)	0.004
OS from CNS mets	74.48 months (95% CI 46.05-NR) 46.74 months (95% CI 31.32-NR) 18.26 months (95% CI 9.61-NR)	43.52 months (95% CI 20.76-47.6) 25.66 months (95% CI 18.88-32.4) 11.51 months (95% CI 2.11-18.26)	115.79 months (95% CI 58.32-NR) 52.96 months (95% CI 35.99-NR) 23.36 months (95% CI 0.49-NR)	0.133

Adenoca NOS, adenocarcinoma not otherwise specified; CI, confidence interval; CNS, central nervous system; CNSmfs, CNS metastasis-free survival; DFI, disease-free interval; Ex pleom. ad., ex pleomorphic adenoma; F, female; HNSCC, head and neck squamous cell carcinoma; M, male; NR, not reached; OS from primary, overall survival measured from the date of diagnosis of primary tumor; OS from R/M, overall survival measured from the date of diagnosis of recurrent/metastatic tumor; OS from CNS mets, overall survival measured from the date of diagnosis of CNS metastases; RT-induced, radiation-induced; SDC, salivary duct carcinoma.

* Fisher’s exact test.

** Mann-Whitney test.

*** log-rank test.

**Figure 1:**
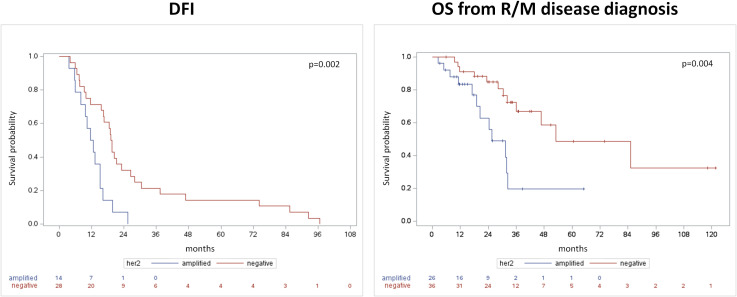
Disease-free interval from primary tumor diagnosis (left panel) and overall survival from the occurrence of recurrent/metastatic disease (right panel).

DFI and OS (both from primary and from R/M) were significantly shorter in HER2-positive SGC patients than in HER2-negative subjects (**Table 1**, **Figure 1**). In patients with HER2-positive disease, HR for disease recurrence (vs. HER-negative) was 2.97 (95% CI 1.44-6.1, p=0.003). HR for death from R/M disease was 3.22 (95% CI 1.39-7.49) in HER2-positive vs. negative SGC patients (p=0.007).

Nineteen patients (31%) did not receive a loco-regional treatment due to the metastatic disease at presentation. In the R/M setting, androgen deprivation therapy (ADT) was administered in 79% of patients overall (89% of HER2-negative vs. 65% of HER-positive patients, p=0.032). An anti-HER2 directed treatment was administered in 27% of HER-positive SGC patients. Systemic targeted treatments were delivered in three HER-negative cases, one within a clinical trial (olaparib followed by palbociclib), two owed to the evidence of actionable tumor mutations (1 tipifarnib for *HRAS* mutation, 1 dabrafenib and trametinib for *BRAF* mutation).

Further details about treatments are reported in **Table 1**.

### 3.2 CNS metastases

Compared with patients with HER2-negative SGCs, the HER2-positive group showed a trend towards higher prevalence of CNS metastases: 40% (10) vs. 24% (9) (p=0.263). Three cases (all HER2-positive) presented with CNS metastases at diagnosis, while in the remaining sixteen cases (7 HER2-positive, 9 HER2-negative) CNS involvement was found at least 6 months after the diagnosis of primary tumor. In these cases, there was a non-statistically significant trend (p=0.083) towards a shorter TTCNS in HER2-positive subjects (**Table 1**). Compared to HER2-negative disease, in HER-positive SGC patients HR for CNSmfs was 2.88 (95% CI 0.87-6.97, p=0.089), and HR for TTCNS was 2.59 (95% CI 0.85-7.87, p=0.094).

The 5-year CNSmfs was 57% in HER2-positive vs. 79% in HER2-negative SGC patients (p=0.08, median CNSmfs not reached in both cohorts).

Independently of HER2 status, no statistically significant difference was found between SGC patients without CNS metastases and those developing CNS disease, in terms of median OS from the diagnosis of primary tumor: 94.31 months (95% CI 47.6-NR) vs 43.52 months (95% CI 24.41-NR), respectively (p=0.125). Further details about outcomes stratified according to both HER2 status and CNS disease are reported in **Supplementary Table 1**.

At bivariate analysis considering HER2 status (positive vs. negative) and CNS disease (occurrence of CNS disease ever vs. never), HER2 maintained an independent prognostic significance (HR for OS from primary 4.34, 95% CI 1.71-11.06, p=0.002) and CNS disease status did not (p=0.198, **Table 2**).

**Table 2:** Bivariate analysis for OS according to HER2 status and presence/absence of CNS disease.

	HR (95% CI)	p value
OS from primary		
HER2-pos (vs. neg)	4.34 (1.71-11.06)	0.002
CNS disease ever (vs. never)	1.76 (0.75-4.14)	0.198
OS from R/M		
HER2-pos (vs. neg)	3.12 (1.33-7.32)	0.009
CNS disease ever (vs. never)	1.63 (0.69-3.85)	0.267

CI, confidence interval; CNS, central nervous system; NR, not reached; OS from primary, overall survival measured from the date of diagnosis of primary tumor; OS from R/M, overall survival measured from the date of diagnosis of recurrent/metastatic tumor.

At diagnosis of CNS disease, all patients were treated with CNS lesions radiotherapy (RT), with the exception of two cases (one HER2-positive and one HER-2 negative) who were diagnosed with CNS metastases during systemic treatment for R/M disease. Of note, the patient with HER-2 amplified SGC was on treatment with ADT, at CNS disease (>5 lesions) onset he was treated with chemotherapy, but eventually died after 2 months. The subject with HER-2 negative disease was diagnosed with 2 brain lesions while on ADT, continued hormone therapy beyond progression, and is still alive with stable CNS disease after 13 months from the diagnosis of CNS metastases, at the time of study analysis.

Descriptive analyses on clinical characteristics, treatments, and outcomes in the different clinical scenarios are reported in **Supplementary Tables 2 and 3**. No statistical analyses were performed due to the limited sample size and number of events in each subgroup.

The majority (68%, n.13) received either stereotactic brain RT (SBRT) or Cyberknife radiosurgery, while 7 cases (37%) received whole brain RT (WBRT).

All the 12 patients developing CNS lesions after metastatic disease (with or without loco-regional SGC at diagnosis) received at least one line of palliative systemic treatment.

After the diagnosis of CNS disease, systemic treatments were administered in 17 cases (89%), while the remaining two cases underwent best supportive care (BSC).

Within the group of 12 patients with unavailable HER2 status only one was found with a CNS metastasis, after 17 months from the diagnosis of the primary SGC. He was treated with two lines of hormone treatment and the single CNS metastasis was treated with Cyberknife radiosurgery.

## 4 Discussion

This retrospective study describes clinicopathological characteristics and outcomes in a cohort of patients with R/M AR-positive SDC and adenocarcinoma NOS according to HER2 status, with a particular focus on CNS metastasis.

HER2 overexpression has been associated with poor prognosis in patients with breast and gastric cancers (18) but its prognostic role remains controversial in SGC.

In line with some prior evidences (14, 15), the current study confirmed HER2 overexpression to be associated with worse outcomes in SGC. A higher risk of recurrence and death was reported in the AR-positive/HER2-positive compared to AR-positive/HER2-negative cohort: DFI and OS (both from primary and from R/M) were significantly shorter (mDFI 12.11 m vs 19.28 m p=0.002; mOS from primary 43.52 m vs 115.79 m p=0.0007; mOS from R/M 25.66 m vs 52.96 m p=0.004 respectively). In patients with HER2-positive disease (vs HER2-negative), HR for disease recurrence was 2.97 (95% CI 1.44-6.1, p=0.003) and HR for death from R/M disease was 3.22 (95% CI 1.39-7.49, p=0.007).

Nevertheless, in other previous studies HER2-overexpression was not related to worse prognosis (19), including a recently published retrospective analysis of 200 patients with SDC and adenocarcinoma NOS treated at the MD Anderson Cancer Center. Although no direct comparisons can be made between retrospective case series published at different Institutions, that study and the present one differ from each other for several reasons. In the US study, only 77% of patients had AR-positive disease, and all disease stages were included (i.e., patients with potentially curable disease at diagnosis or with metastatic disease upfront). In our case series, we included patients with R/M only, not all comers, because only those subjects are treated with antiandrogen or anti-HER2 agents, with the exception of patients receiving off label adjuvant therapy after loco-regional treatments.

Moreover, in the MD Anderson study tumors were classified as HER-2 positive if they scored as 2+ or 3+ by IHC, regardless of FISH results (20). In our study, we considered only patients with R/M AR-positive disease and tumors were classified as HER2-positive only if IHC score was 3+ or 2+ with gene amplification by FISH. Even the threshold for AR-positivity was different in the two studies (positivity defined as IHC staining in ≥ 10% of tumor cells in that study versus a combined expression score obtained by summing the scores of staining intensity and extent in our dataset) selecting in our study a population with higher expression of AR and HER2. Finally, a lower prevalence of patients treated with anti-HER2 was observed in our cohort (27%) when compared to the MD Anderson experience. Indeed, in the cited article, more than half (17/32) of HER2-positive SGC patients requiring a first-line systemic therapy were treated with at least one line of anti-HER2-based regimen (10 in first line) (20). Although no direct comparisons can be made between that retrospective series and ours, it is likely that the different regulatory and reimbursement agencies and laws between the US and European Countries might have impacted on this different prevalence.

Thus, the difference between groups with/without AR/HER2 molecular alterations might have become more evident in the present study population. Furthermore, in breast cancer, AR was shown to play an important role in promoting the growth of HER2-positive disease by a functionally significant cross-talk with the HER2 signaling (21).

In our study, a non-statistically significant trend towards a higher risk of CNS metastasis, with shorter CNSmfs (HR 2.88 [95% CI 0.87-6.97]; p=0.089) emerged in HER2-positive compared to HER2-negative cohort. As expected, regardless of HER2 status, the outcomes of patients with CNS metastases were worse than those observed in those without CNS metastases (mOS 43.52m vs 94.31m, although not significant, p=0.125). Nevertheless, after adjustment according to HER2 status, the presence of CNS metastases did not seem to be prognostic *per se*, since this variable lost its prognostic significance at bivariate analysis, while HER2 status did not. This might be explained by the worse prognosis of HER2-amplified SGCs, as known from the literature and as observed in our series. In fact, in the case of neurological involvement, TTCNS (p=0.083) and OS (p=0.133) were shorter, though not significant, also in the HER2-positive group.

It should be noted that only 27% of HER2-positive patients received an anti-HER2 directed treatment, as most patients were treated from 2010 to 2021 when none of these drugs were available for use in clinical practice in Italy. This finding confirms poor outcomes in HER2-positive SGC patients especially if not treated with targeted therapy, as we assume that an additional benefit in clinical outcomes is to be expected by a targeted agent as suggested by recent data (8, 9, 11–13).

In our analysis, there were no statistically significant differences in the clinical characteristics between HER2-positive and HER2-negative populations, but we found a trend for HER2-positive to be younger, less frequently affected by adenocarcinoma NOS, and more frequently affected by CNS metastases. This latter finding resembles the clinical behavior of HER2-positive breast cancer, as confirmed by a recent study of the Unicancer Epidemiological Strategy and Medical Economics (ESME) metastatic breast cancer (MBC) database (n = 16,701): 24.6% of the patients developed brain metastasis, and the risk was higher for patients with HER2-positive/hormone receptor (HR)- negative and triple-negative (TNBC) breast cancer (22).

All these findings confirm the importance of assessing the HER2 status always at diagnosis of SDC and adenocarcinoma NOS, because of its prognostic role, and it should guide the treatment choice, as recommended by major guidelines (23, 24).

With the limit of a small sample size, we observed a 8% prevalence of CNS metastases even in the group with unavailable HER2 status. This underlines the importance to assess a prompt CNS staging at diagnosis in HER2-positive disease and during the follow-up in any AR-positive SGC, even if it does not seem to impact prognosis per se in our bivariate analysis. Further studies are needed to confirm this suggestion, similarly to the current studies on this subject in breast disease. In fact, despite compelling evidence, in HER2 breast cancer, the upfront screening for CNS disease is currently not recommended, due to a lack of data supporting its benefit in terms of overall survival (25). Therefore, the potential benefit from proactive screening strategies in selected patients with increased risk for CNS metastases is being studied in ongoing clinical trials (NCT03881605, NCT03617341, NCT04030507).

In our study, only patients with AR-positive disease were studied, and in this setting the HER2 status was prognostic. This points out the need for more effective treatments for patients with SGC harboring both AR and HER2 overexpression. A retrospective study on SDC or AR-positive adenocarcinoma NOS reported an objective response rate (ORR) to ADT of 55% in the first-line setting and 16.7% for subsequent lines (26), suggesting that ADT as first-line therapy provides a relevant clinical benefit in this setting. Given the encouraging activity in HER2-positive SGC with HER2-targeted therapies (8–10, 12, 27), we can speculate that adding HER2-blockade to ADT may improve survival outcomes. In this scenario, interestingly, recent *in vitro* experiments showed that enzalutamide, an anti-androgen drug, inhibits the growth of HER2 breast cancer cells (21). This suggests that the activity of AR inhibition might be anticipated in HER2-positive SGCs, even independently of HER2 inhibitors. Clinical trials focused on AR-positive/HER2-positive SGC patients are needed to evaluate this suggestion.

There are some study limitations that need to be considered. This is a retrospective study performed at a single institution. Moreover, in 16% of the case series HER2 status was not available, and this may have produced a bias in the analyses. This lack of information in 12 subjects is mainly due to the fact that this case series was analyzed by collecting consecutive patients treated at our Institution from 2010 to 2021, and the first robust evidence of the activity of anti-HER2 agents in HER2-amplified R/M SGC patients was published in 2019. Therefore, at the time the first patients included in this article were on treatment at our Institution, neither trastuzumab nor any other anti-HER2 drugs were formally approved yet for R/M SGC patients worldwide.

A strength of this study is the number of patients, which is significant for a monocentric cohort of patients with recurrent/metastatic disease only affected by such a rare tumor. Collaborative ongoing efforts such as EURACAN could provide a platform to further investigate these rare entities. Furthermore, only a minority of HER2-positive patients received anti-HER2 drugs, which are known to have an impact on the response of CNS metastases.

## 5 Conclusions

This study focuses on CNS metastases in SDC and adenocarcinoma NOS patients, suggesting possible connections with HER2 status. Further studies are needed to confirm our findings and to investigate the clinical benefit of tackling the two biological pathways in patients affected by these rare and aggressive malignancies.

## Data availability statement

Since the data of this article include sensitive data, they will be made available upon reasonable request to the corresponding Author.

## Ethics statement

The studies involving human participants were reviewed and approved by Fondazione IRCCS Istituto Nazionale dei Tumori, Milan, Italy. Written informed consent for participation was not required for this study in accordance with the national legislation and the institutional requirements.

## Author contributions

SC and LL contributed to conception and design of the study. PQ reviewed all histological specimens. All authors contributed to data collection and curation. SC organized the database and performed the statistical analysis. SC and IN wrote the first draft of the manuscript. SC, IN, LL, CR, and AO wrote sections of the manuscript. All authors contributed to the article and approved the submitted version.
