# Supplementary material for: HER2 status in recurrent/metastatic androgen receptor overexpressing salivary gland carcinoma patients
Source: Front Oncol. 2023 Jan 17;12:1096068. doi: 10.3389/fonc.2022.1096068 (PMC9887140; doi:10.3389/fonc.2022.1096068)
Supplement: Supplementary file 1 [file Table_1.docx]

Supplementary Material

# Supplementary Tables

| 5-year survival | HER2-positive | | HER2-negative | | p value |
| --- | --- | --- | --- | --- | --- |
|  | Never CNS disease  (n=16) | CNS disease ever  (n=10) | Never CNS disease  (n=27) | CNS disease ever  (n=9) |  |
| OS from primary | 30.5% | 0% | 68.3% | 71.4% | 0.002 |
| OS from R/M | 33.4% | 0% | 47.9% | 52.5% | 0.012 |

Supplementary Table 1. Survival stratified according to HER2 status and presence/absence of CNS disease.

Abbreviations: CI, confidence interval; CNS, central nervous system; NR, not reached; OS from primary, overall survival measured from the date of diagnosis of primary tumor; OS from R/M, overall survival measured from the date of diagnosis of recurrent/metastatic tumor.

|  | LR 🡪 CNS | | | LR 🡪 M1 🡪 CNS | | |
| --- | --- | --- | --- | --- | --- | --- |
| Timing of CNS disease | Overall  (4 pts) | HER2+  (2 pts) | HER2-  (2 pts) | Overall  (6 pts) | HER2+  (3 pts) | HER2-  (3 pts) |
| N. lesions  1  2-4  ≥5  RT to CNS  Whole brain  Cyberknife  SBRT  Systemic treatments after CNS  Lines  0  1  2-3  ≥4  Treatments  ADT  CT  Anti-HER2  Systemic treatments before CNS  Lines  1  2-3  ≥4  Treatments  ADT  CT  Anti-HER2  LR treatment  Surgery-RT  Surgery-CRT | 2  1  1  4  1  -  3  4  1  3  -  -  2  3  1  -  4  2  2 | 1  1  -  2  -  -  2  2  -  2  -  -  1  2  1  -  2  1  1 | 1  -  1  2  1  -  1  2  1  1  -  -  1  1  -  -  2  1  1 | -  2  4  4  2  1  2  6  -  4  2  -  5  2  1  6  4  2  -  4  4  -  6  3  3 | -  -  3  2  1  1  -  3  -  2  1  -  2  1  1  3  3  -  -  1  2  -  3  1  2 | -  2  1  2  1  -  2  3  -  2  1  -  3  1  -  3  1  2  -  3  2  -  3  2  1 |
| Median DFI (95% CI)  Median TTCNS (95% CI)  Median interval from RM to CNS (95% CI)  Median OS from primary (95% CI)  Median OS from RM (95% CI)  Median OS from CNS (95% CI) | -  15.4 m (5.9-NR)  -  115.8 m (NR)  -  23.3 m (NR) | -  9.2 m (5.9-NR)  -  NR  -  NR | 55.4 m (18.5-NR)  -  115.79 m (NR)  -  23.4 m (NR) | 12.1 m (7.3-NR)  22.6 m (17.4-NR)  10.7 m (5.4-NR)  43.5 m (20.1-NR)  31.9 m (7.5-NR)  20.3 m (2.1-NR) | 12.7 m (11.5-NR)  24.6 m (18-NR)  8.4 m (5.4-NR)  43.5 m (20.1-NR)  32 m (7.5-NR)  11.5 m (2.1-NR) | 9.3 m (7.3-NR)  20.6 m (17.4-NR)  11.3 m (10.1-NR)  NR (37.8 m -NR)  NR (30.5 m -NR)  NR (20.3 m -NR) |

Supplementary Table 2. CNS metastases in patients diagnosed with loco-regional disease

Abbreviations

LR 🡪 CNS: CNS metastases as first disease recurrence after treatment for loco-regional disease; LR 🡪 M1 🡪 CNS: CNS metastases as subsequent recurrence after palliative treatments for R/M disease diagnosed after treatment failure for loco-regional primary disease; CI: confidence interval; CM: carcinomatous meningitis; CRT: chemoradiation; LR: loco-regional; m: months; N: number; NR: not reached; pts: patients; R/M: recurrent/metastatic; RT: radiotherapy.

|  | Upfront CNS disease | | | M1 🡪 CNS | | |
| --- | --- | --- | --- | --- | --- | --- |
| Timing of CNS disease | Overall  (3 pts) | HER2+  (3 pts) | HER2-  (no pts) | Overall  (6 pts) | HER2+  (2 pts) | HER2-  (4 pts) |
| N. lesions  1  2-4  ≥5  CM  RT to CNS  Whole brain  Cyberknife  SBRT  Systemic treatments after CNS  Lines  0  1  2-3  ≥4  Treatments  ADT  CT  Anti-HER2  Systemic treatments before CNS  Lines  1  2-3  ≥4  Treatments  ADT  CT  Anti-HER2 | -  1  2  -  3  1  2  -  3  -  -  2  1  3  2  2  - | -  1  2  -  3  1  2  -  3  -  -  2  1  3  2  2  - | - | 3  1  1  1  6  3  2  3  6  1  3  2  -  4  2  1  6  2  4  -  4  5  1 | 1  1  -  -  2  1  2  1  2  -  1  1  -  1  1  1  2  1  1  -  1  2  1 | 2  -  1  1  4  2  0  2  4  1  2  1  -  3  1  -  4  1  3  -  3  3  - |
| TTCNS  Median OS from RM (95% CI)  Median OS from CNS (95% CI) | -  -  18.3 m (4.9-NR) | -  -  18.3 m (4.9-NR) | -  -  - | 23.4 m (12-NR)  32.1 m (24.4-NR)  12.2 m (0.5-NR) | 16.9 m (14.8-NR)  27.9 m (24.4-NR)  10.9 m (9.6-NR) | 28.4 m (12-NR)  NR (32 m - NR)  NR (0.5 m - NR) |

Supplementary Table 3 - CNS metastases in patients diagnosed with upfront metastatic disease

Abbreviations

M1 🡪 CNS: CNS metastases after metastatic disease at diagnosis; CM: carcinomatous meningitis; CRT: chemoradiation; LR: loco-regional; N: number; NR: not reached; pts: patients; R/M: recurrent/metastatic; RT: radiotherapy.
